# Genome-wide identification and characterization of bZIP transcription factors and their expression profile under abiotic stresses in Chinese pear (*Pyrus bretschneider*i)

**DOI:** 10.1186/s12870-021-03191-3

**Published:** 2021-09-09

**Authors:** Muhammad Aamir Manzoor, Muhammad Mudassar Manzoor, Guohui Li, Muhammad Abdullah, Wang Han, Han Wenlong, Awais Shakoor, Muhammad Waheed Riaz, Shamsur Rehman, Yongping Cai

**Affiliations:** 1 School of Life Sciences, Anhui Agricultural University, Hefei, 230036 China; 2 Department of Software Engineering, University of Gujrat, Lahore, 54000 Pakistan; 3 Department of Environment and Soil Sciences, University of Lleida, Avinguda Alcalde Rovira Roure 191, 25198 Lleida, Spain; 4 College of Agronomy, Anhui Agricultural University, Hefei, 230036 China; 5 Co-Innovation Center for Sustainable Forestry in Southern China, Key Laboratory of Forest Genetics & Biotechnology, Ministry of Education, College of Biology and the Environment, Nanjing Forestry University, Nanjing, China

**Keywords:** *PbbZIP* gene family, Phylogenetic analysis, Expansion, Expression analysis, Stress response

## Abstract

**Background:**

In plants, basic leucine zipper transcription factors (TFs) play important roles in multiple biological processes such as anthesis, fruit growth & development and stress responses. However, systematic investigation and characterization of bZIP-TFs remain unclear in Chinese white pear. Chinese white pear is a fruit crop that has important nutritional and medicinal values.

**Results:**

In this study, 62 bZIP genes were comprehensively identified from Chinese Pear, and 54 genes were distributed among 17 chromosomes. Frequent whole-genome duplication (WGD) and dispersed duplication (DSD) were the major driving forces underlying the bZIP gene family in Chinese white pear. bZIP-TFs are classified into 13 subfamilies according to the phylogenetic tree. Subsequently, purifying selection plays an important role in the evolution process of *PbbZIPs*. Synteny analysis of bZIP genes revealed that 196 orthologous gene pairs were identified between *Pyrus bretschneideri*, *Fragaria vesca, Prunus mume,* and *Prunus persica*. Moreover, *cis*-elements that respond to various stresses and hormones were found on the promoter regions of *PbbZIP*, which were induced by stimuli. Gene structure (intron/exon) and different compositions of motifs revealed that functional divergence among subfamilies. Expression pattern of *PbbZIP* genes differential expressed under hormonal treatment abscisic acid, salicylic acid, and methyl jasmonate  in pear fruits by real-time qRT-PCR.

**Conclusions:**

Collectively, a systematic analysis of gene structure, motif composition, subcellular localization, synteny analysis, and calculation of synonymous (Ks) and non-synonymous (Ka) was performed in Chinese white pear. Sixty-two bZIP-TFs in Chinese pear were identified, and their expression profiles were comprehensively analyzed under ABA, SA, and MeJa hormones, which respond to multiple abiotic stresses and fruit growth and development. *PbbZIP* gene occurred through Whole-genome duplication and dispersed duplication events. These results provide a basic framework for further elucidating the biological function characterizations under multiple developmental stages and abiotic stress responses.

**Supplementary Information:**

The online version contains supplementary material available at 10.1186/s12870-021-03191-3.

## Background

Transcription factors (TFs) play crucial roles in the regulation and biological processes under various environmental stress [1, 2]. The basic leucine zipper (bZIP) transcription factors family member is one of the most diverse and largest transcription factors in eukaryotes [3]. The length of the domain is 60–80 amino acids containing two regions with various functions; the basic region is highly conserved and consists of 16 amino acid residues with specific DNA binding (N-× 7-R/K-× 9) [4]. On the other hand, the leucine zipper is less conserved and composed of several helical structures, which are responsible for dimerization stability and specificity (homo/heterodimerization) [5].

Abiotic stress resistance in plants is mediated by the hormone ABA, which regulates many stress-responsive genes and confers abiotic stress tolerance in plants [6]. The group A *AtbZIP* protein includes ABA-responsive element-binding proteins (AREB) or ABRE binding factors (ABF) that have been functionally identified as major regulators of ABA-dependent gene expression and the abiotic stress response [1, 6]. Phosphorylation of category III SnRK2s activates *AREB1/ABF2, AREB2/ABF4, ABF1*, and *ABF3* in Arabidopsis, controlling the expression of their downstream genes [7, 8]. *AtbZIP17, AtbZIP60, and AtbZIP28* proteins play significant roles in ER stress responses [9]. In Arabidopsis *AtbZIP28, AtbZIP17* genes are activated by salt stress and function as a salt stress sensor/transducer [10]. Salt treatment increased the transcription of *AtbZIP1, MtbZIP2*, and *MtbZIP26* from *A. thaliana* and *Medicago truncatula*, resulting in an increase in salt stress resistance [11–13]. The *bZIP* family members have been comprehensively identified and functionally characterized in various plants such as *A. thaliana* (75 genes) [1], *B. napus* (247 genes) [14], *Solanum lycopersicum* (69 genes) [15], *B. distachyon* (96 genes) [16], *Oryza sativa* (89 genes) [3], *Zea mays* (125 genes) [17], *Glycine max* (131 genes) [18]*, Coniothyrium minitans*, (34 genes) [19], *Vitis vinifera* (55 genes) [20], *Malus domestica* (116 genes) [21], *Fragaria vesca* (50 genes) [22], *Sorghum bicolor* (92 genes) [23] and *Prunus persica* (47 genes) [24], *Ricinus communis L*. [25].

Plant bZIP proteins plays important roles in plant growth and diverse biological functions, including somatic embryogenesis [26], seed germination [20, 27], cell elongation [28], vascular development [29], floral induction and growth [30], unfolded protein response [31], and photomorphogenesis [32]. Plant bZIP transcription factors have also been involved in a variety of abiotic/biotic stress-related including hormone signaling and sugar response [33–36], light response [37, 38], pathogen infection [39, 40], cold signaling [41, 42], osmotic stresses [43], heat stress [44] and so on. Generally, ABA-responsive binding factor (ABRE) and binding protein (AREB) are associated with ABA and stress-related factors [45]. In rice, *OsbZIP71* acts as ABA-mediated salt and drought tolerance [46]. *ZmbZIP17* in maize is also highly induced with the interaction of ABA (*cis*-element ABRE) [45]. *SlbZIP33* (SlAREB1) was implicated in stress-induced responses in tomato and acted as critical components in regulating the expression of important metabolic pathway-related genes and metabolic programming during fruit ripening [47, 48].

Chinese pear (*Pyrus. bretschneideri*) fruit crop is cultivated worldwide, especially in China, and has become most popular or essential due to its high medicinal and nutritional value [49, 50]. Although bZIP members have been identified in various model plants, this gene family has not been comprehensively studied in *Pyrus. bretschneideri*. However, a comprehensive and systematic analysis of the bZIP family has been identified in Chinese pear. Finally, in this study, 62 *PbbZIP* genes were identified and classified into 13 groups according to the phylogenetic tree. The evolutionary relationship/history, chromosomal distribution, conserved domain, gene duplication, gene structure analysis, conserved motif, microsynteny relationship, *cis*-elements on the promoter regions, ka/ks value analysis, and other important information were investigated in *PbbZIP* genes. The expression profiles of *PbbZIP* genes in different fruit development stages were analyzed using RNA-seq data. Afterward, the expression patterns of 12 genes were examined in response to Abscisic acid (ABA), salicylic acid (SA), and methyl salicylate (MeJA) by conducting qRT-PCR. These results would provide specific knowledge and evolution in Chinese pear for future studies.

## Results

### Identification of bZIP gene family in Chinese white pear

The pear bZIP transcription factors family members were identified by using Hidden Markov Model (HMM) and Blast method. A systematic approach bZIP domain (PF00170) analysis was also performed using the Pfam database. Initially, pear bZIP family members were identified, and their sequences were downloaded from the pear genome. Finally, identified candidate genes were briefly analyzed and verified the bZIP domain. Subsequently, 62 members of this family encoding complete bZIP DNA binding domain were selected for sequence alignment and for Further analysis. Nomenclature of these identified genes was given based on chromosome position and renamed all genes *PbbZIP1* to *PbbZIP62* (Table S2). The length of amino acid varied from 141 AA (*PbbZIP41*) to 1726 AA (*PbbZIP16*), with a cross-ponding isoelectric point (IP) varied from 4.96 (*PbbZIP28*) to 10.51 (*PbbZIP50*). The molecular weight of these genes ranged from 16,313.4 kDa to 194,044.9 kDa. Detailed information, including gene i.d, given name, molecular weight, isoelectric point, gene size, and chromosomal distribution, are mentioned in Table S2.

### Phylogenetic analysis

**Fig. 1:**
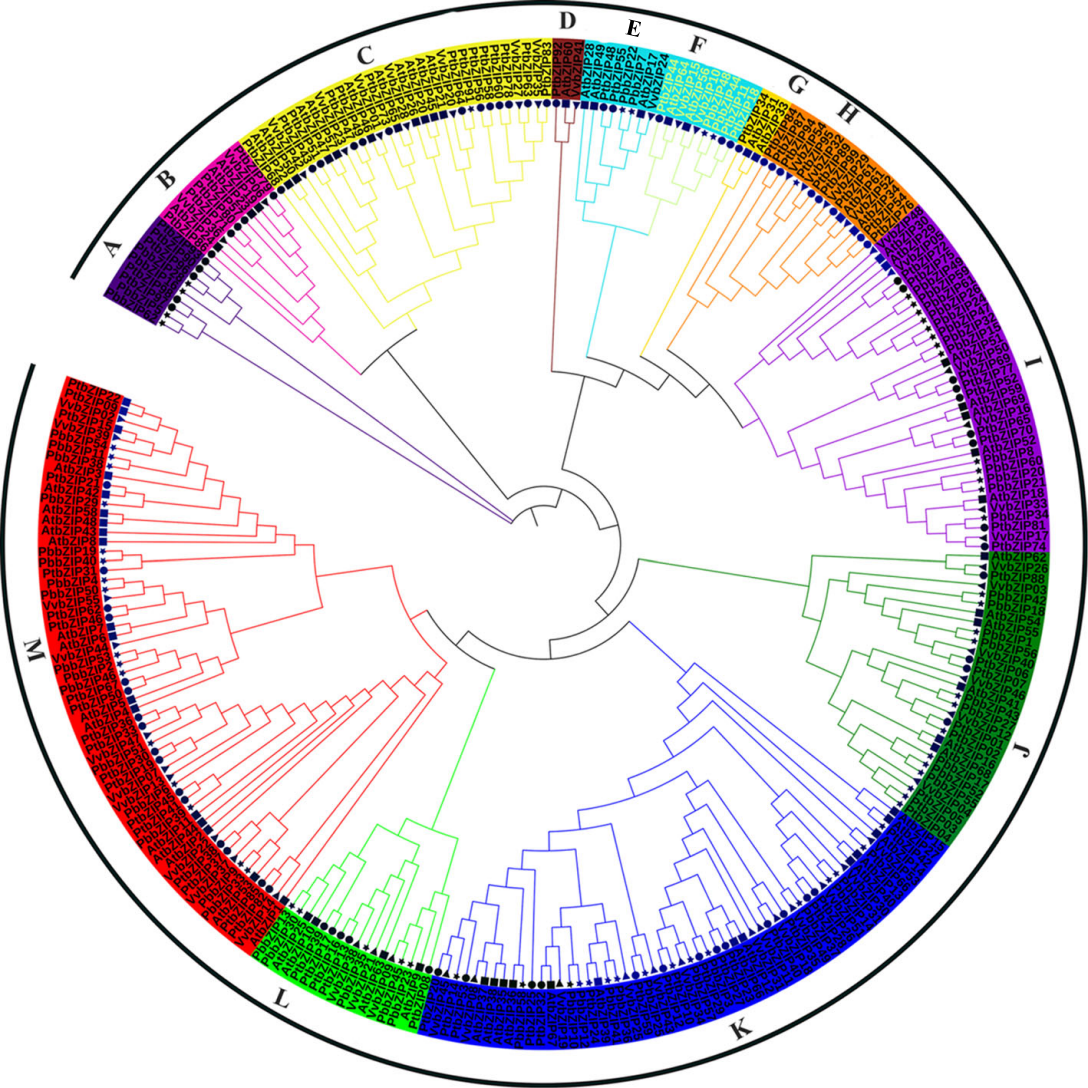
The phylogenetic tree of bZIP genes in Chinese pear, Arabidopsis, grapes, and poplar. Different color indicates different subfamilies (A-M)

To further understand the evolutionary relationship, difference, and similarities of bZIP transcription factor between pear (62 genes), Arabidopsis (75 genes), grapes (55 genes), and poplar (86 genes). The unrooted phylogenetic tree was generated through neighbor-joining methods (NJ-M), and the maximum likelihood method (ML-M). For future study neighbor-joining tree was used to perform a phylogenetic characterization in Chinese pear with Arabidopsis (Fig. 1 and S2). Finally, the phylogenetic tree was visualized through itol software and categorized into 13 subfamilies (A, B(F/*Ara*), C(D/*Ara*), D, E(B/ *Ara*), F(H/*Ara*), G, H(E/*Ara*), I(I/Ara), J(G/*Ara*), K(A/*Ara*), L(C//*Ara*), M(S/*Ara*). These groups were classified according to the Arabidopsis groups, in subfamily D and G absent in pear, but in the subfamily, A only contained pear and poplar genes except Arabidopsis may be due to functional divergence or these subfamilies indicating that specific functions. Furthermore, an inspection of the phylogenetic tree, maximal *PbbZIPs* members contained in subfamily A (13) and minimal in subfamily E-F (2) (Fig. 1). Subfamily C, D, G, and H formed clades without pear genes indicating that these clades might be specific for poplar and Arabidopsis. Gene loss and gain mechanisms occurred in evolutionary mechanisms and caused functional divergence.

### Gene ontology (GO) analysis and subcellular localization of *PbbZIP*

**Fig. 2:**
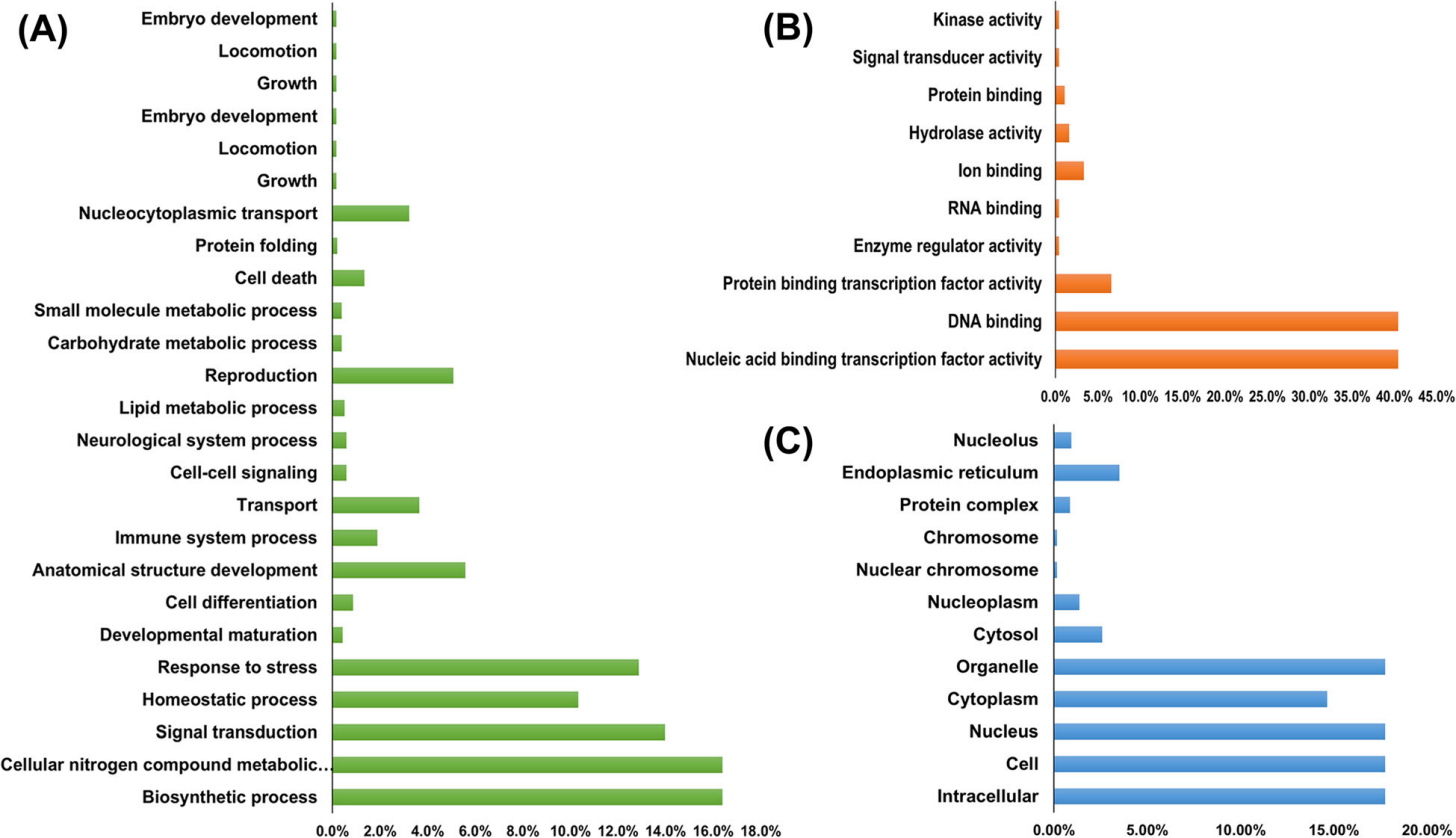
Predicted molecular functions, cellular components, and biological processes of the *PbbZIP* gene family

GO annotation analysis was performed for the prediction of various functions in *PbbZIP* members. Gene ontology such as cellular components, biological processes, and molecular functions. Additionally, 62 *PbbZIP* proteins were grouped into 44 functional groups according to protein similarities under the three basic groups. In the ontology of biological process, we evaluate that the highest percentage were involved in the biosynthetic process (16.39%) and cellular nitrogen compound metabolic process (16.39%). Among the *PbbZIP*, 13.98, 12.87, 10.33% of genes showed potential involvement in signal transduction, response to stress, and hemostatic process, respectively. Also, some genes are involved in development (0.43%), the immune system 1.88%, cell deafferentation 0.86%, and anatomical structure 5.50% (Fig. 2). In the ontology of molecular functions, *PbbZIPs* genes the highest percentage are involved in nucleic acid binding transcription function activity, and DNA binding 40.46%, followed by protein binding transcription factor 5.59%, enzyme regulator activity 0.40%, RNA binding 0.40%, ion binding 3.36%, hydrolase activity 1.61%, protein binding 1.80%, signal transducer activity 0.40%, kinase activity 0.40%.

Moreover, cellular component ontology suggested that also contain cellular components followed as intracellular, cell, and organelle found are same and high percentage 17.74% as compared to another cytoplasm (14.65%), cytosol (2.58%), nuclear chromosome (0.16%), nucleoplasm (1.36%), chromosome (0.16%), protein complex (0.86), and endoplasmic reticulum (0.92%) (Table S3). Subcellular localization was predicted by using CELLOGO tool software. These results indicate that most of the *PbbZIP* genes (96.8%) were localized in nuclear, while remained genes were involved in cytoplasm and chloroplast (Table S3).

### Gene structure analysis and identification of conserved motif

**Fig. 3:**
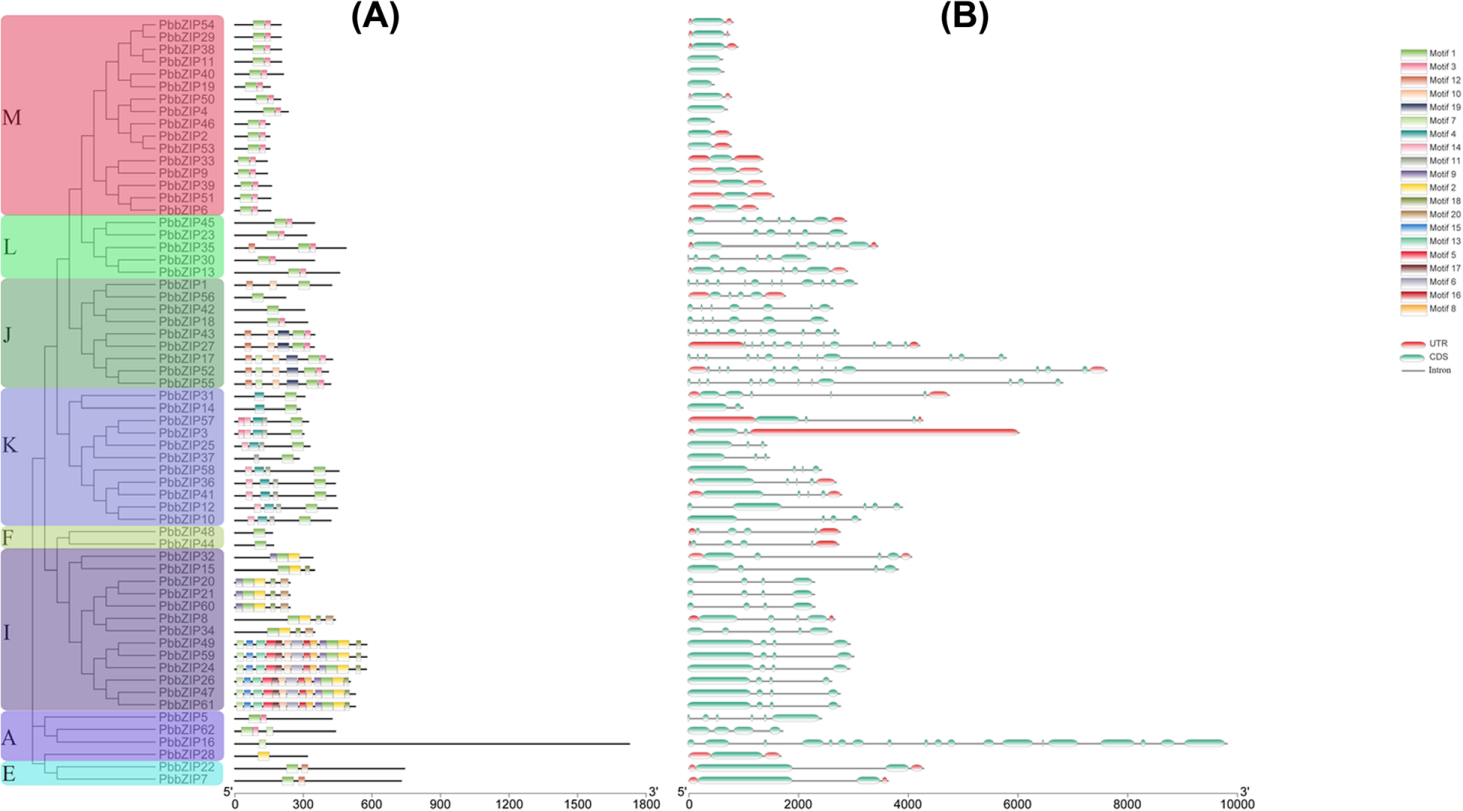
Distribution of introns-Exons and Conserved motifs of the bZIP gene family in Chinese pear. (**A**) Phylogenetic relationship and numbers of exons/introns of a gene family in Chinese pear. (**B**) location of Conserved motifs on each gene

Structural analysis was performed by comparing general feature format (GFF3) files of each bZIP gene in Chinese white pear to understand the phenomena of structural diversity. The composition and position of introns/exons identified using Gene structure display server and compare the gene structure among different or same subfamily. In *PbbZIP* genes contain maximum introns/exons (18/19) (Fig. 3A). These results revealed that 5 *PbbZIP* genes (*PbbZIP11*, 40, 19, 4, and 46) had no introns in their gene structures, while remained *PbbZIP* genes contained 1 to 18 introns.

Additionally, the *PbbZIPs* gene in subfamily A contained the largest number of exons/introns; for example, *PbbZIP16* had 19-exons/18-introns. On the other hand, the number of introns/exons variant also in the same subfamily, such as *PbbZIP31,* had 4-exons/4-introns. These results revealed that the divergence of introns/exons structure in the *PbbZIPs* family might be related to different functions.

Conserved motif analysis was performed through the protein sequence of *PbbZIP* genes using a MEME server. Distribution and composition of conserved *PbbZIP* motif ratio similar among the subfamily, supporting the results of the phylogenetic tree. A total of twenty (20) motifs were predicted in *PbbZIP* genes, and motif contained in *PbbZIP* members varied within the subfamily. *PbbZIP* genes were divided into different subfamilies (M, L, J, K, F, I, A, and E) according to the phylogenetic tree and divergence of motifs. The subfamily-I encoded a large number of conserved motifs. The number of motifs was variant among different and same subfamilies. The number of motifs was different between these subfamilies M (maximum number of motifs 2), L (maximum number of motifs 3), J (number of motif 9), K (maximum number of motif 6), F (maximum number of motif 1), I (maximum number of motif 13), A (maximum number of motif 3) and E (maximum number of motif 2) (Fig. 3B). Conserved motif two highly contribution is mostly genes. The presence of numerous motifs in *PbbZIP* members indicates that similar duplication events. In Chinese pear (*Pyrus bretschneideri*), among 65 bZIP members, 50 residues conserved domain (motif 2). The presence of a conserved motif between the same bZIP protein, especially within the subfamily, likely reflects the similarities in functions. These proteins conserved different motifs in the same subfamily reflect that they have different functions (Table S4).

### Identification of gene duplication events, expansion, and chromosomal distribution

**Fig. 4:**
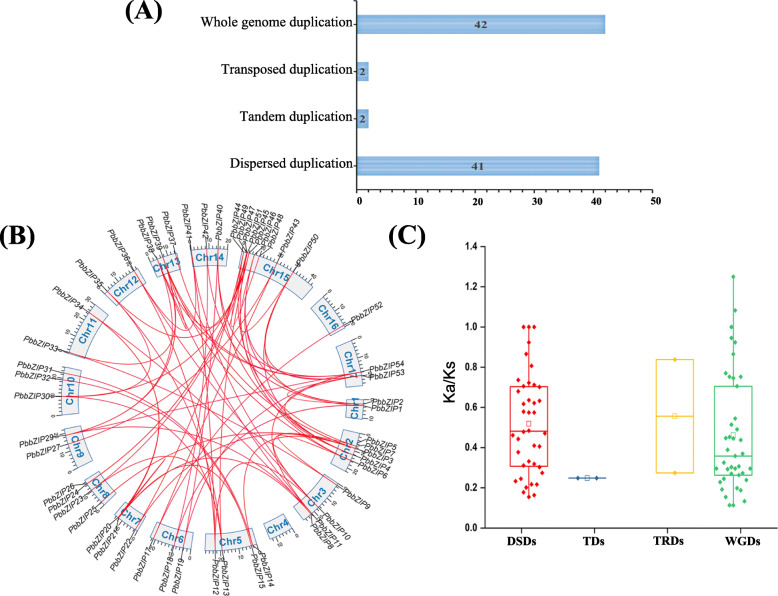
Gene duplication events (WGD, TD, TRD, and DSD) and localization of *PbZIP* gene family in *P. bretschneideri*. (**A**) The different number represents the number of duplications in each event (**B**) duplicated gene pairs localized on the different chromosomes and connected with a red line. (**C**) Estimated ka/ks values indicate that different gene duplications events (DSD, WGD, TD, and TRD)

To elucidate the origin of the bZIP TFs gene family in Chinese pear, four types of duplication (WGD-whole genome duplication, TD-tandem duplication, TRD-transposed duplication, and DSD- dispersed duplication) were carryout (Fig. 4B). A total of 87 duplicated pairs were reported in Chinese pear followed by DSDs (41 gene pairs), WGDs (42 gene pairs), TDs (2 gene pairs), and TRDs (2 gene pairs), indicating that the expansion of the gene family (Fig. 4A). Expansion of gene family basically occurred by the whole genome duplication and dispersed duplication [51]. In the study, identified tandem and dispersed duplication to elucidate expansion of bZIP genes in *Pyrus bretschneideri*. Simultaneously, these results also indicate the complicated duplication mechanism of the *PbbZIP* family. All duplication modes (DSDs, WGDs, TDs, and TRDs) were involved in the evolution and expansion of *PbbZIP* genes. In pear, 41% of genes showed dispersed duplication (DSD), while 2% tandem duplication (TD) suggesting that dispersed duplication events highly contribute to the expansion and evolution of the *PbbZIP* genes family as compared to tandem duplication events (Table S5). The number of duplicated genes was different on duplication mode such as WGD (maximum eight duplicated genes on chromosome 15), DSD (maximum six duplicated genes on scaffold), TD (maximum one duplicated genes on chromosome 15 and scaffold respectively), TRD (maximum one duplicated genes on chromosome 8 and scaffold respectively) (Table S5). To understand the localization of *PbbZIP* genes on a different chromosome number, all genes of pear are mapped except that gene located on scaffold according to the pear genome. As a result of the 62 *PbbZIP* gene, 54 genes were located on the chromosome, and eight genes were located on the scaffold. Moreover, maximum 9 genes are traced on chromosome 15. On the other hand, chromosome 16 minimum carried one gene (Fig. S1 and Table S2).

### Estimation of Ka/Ks analysis

To determine the evolutionary date in gene duplication events, we calculate synonymous (Ks) and non-synonymous (Ka) values for 87 duplicated gene pairs. The Ka/Ks value ranged from 1.24 to 0.21, as shown in Table S5. In general, Ka/Ks value greater than one was suggested that positive selection, Ka/Ks less than 1 suggested purifying selection, and Ka/Ks =1 suggest neutral selection [52, 53]. In our study, all *PbbZIP* gene pairs Ka/Ks value less than 1, indicating that these genes are primarily undergone in purifying selection. On the other hand, four genes ka/ks value is equal to 1, indicating that positive selection (Fig. 4C). We also calculated the ka/ks value in DSD, TD, TRD, and WGD. These gene pairs *PbbZIP40-PbbZIP19* had (ka/ks 1.0827), and *PbbZIP10- PbbZIP12* (ka/ks 1.2498) had higher Ka/Ks value indicates that this gene family complicated evolutionary history.

### Synteny analysis of *PbZIP* genes

**Fig. 5:**
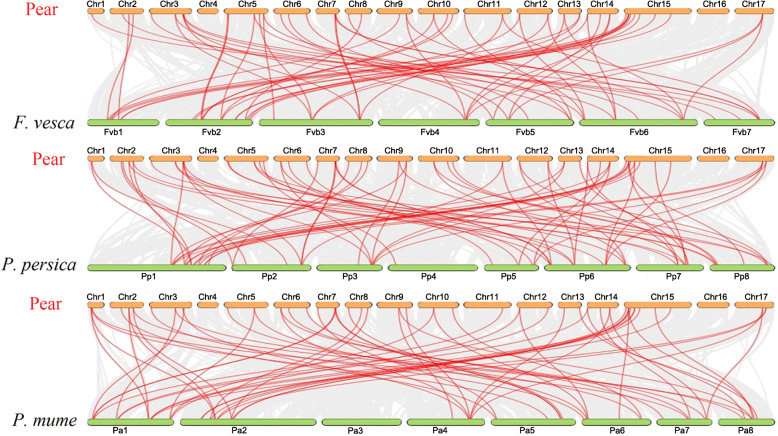
Synteny relationship of *Pyrus bretschneideri*, *Fragaria vesca, Prunus mume,* and *Prunus persica* and the red color line represents in four Rosaceae species

The collinearity relationship is constructed among homology in other species to illustrate the collinearity association of *PbZIP* genes between *Fragaria vesca*, *Prunus mume*, and *Prunus persica*. The collinearity relationship between *Pyrus bretschneideri*, *Fragaria vesca, Prunus mume,* and *Prunus persica* showed a total of 196 orthologous pairs. The orthologous pairs of *PbbZIP* genes with other species, including *Pyrus bretschneideri*-*Fragaria vesca* (63 pairs), *Pyrus bretschneideri-Prunus mume* (61 pairs), and *Pyrus bretschneideri-Prunus persica* (72 pairs) Table S6. Finally, these results indicate that the close similarities relationship with other orthologous and phylogenetic relationships (Fig. 5).

### *Cis*-elements analysis

**Fig. 6:**
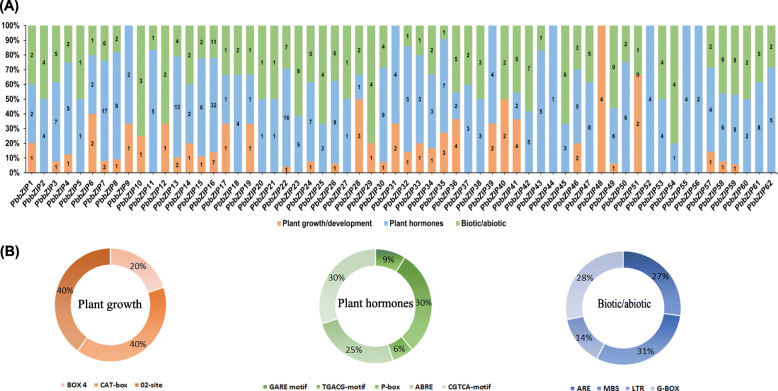
(**A**) The number of *cis*- Acting elements of *PbZIP’s* gene and divided into three groups (Biotic/abiotic, Phytohormones, and Plant growth/development). The X. axis and Y. axis display the *PbZIP* genes and percentage of *cis*-elements, respectively. (**B**) The pie charts showed that the percentage of each *cis*-elements in each group

Promoter activities play a vital role in the regulatory mechanism and different types of gene functions [54]. The *cis*-elements analysis on promoter regions was analyzed to estimate the metabolic network, including stress response, tissue-specific expression, multi stimulus-response, and other environmental conditions [53, 55]. Trans-acting elements and *cis*-regulatory elements play a potential mechanism for the increase and decrease of the expression of any gene. *Cis*-elements were obtained on the promoter regions of *PbbZIP* genes to understand the potential role and mechanism. We observed the highest amount of *cis*-elements on the promoter regions through plantCare database. Furthermore, *cis*-elements were classified into three basic categories: (1) stress response (biotic/abiotic) (2) plant growth/development (3) phytohormones responsive (Fig. 6). In-plant growth/development, mostly *PbbZIPs* genes contain as regulatory elements such as Box 4, MRE, G-Box (plant growth response in light), CAT-box (meristem related), 02-site (zein metabolism related). In stress related *cis*-elements including MBS (light responsive), LTR (low temperature related) and ARE (responsible anaerobic induction). Subsequently, in phytohormones related class identified large number of *cis*-elements including P-box and GARE-motif (gibberellin response elements). Among all these three classes (stress response, plant growth/development, phytohormones responsive) the dominant portion G-box (28%), CAT-box and 02-site (40%) and TGACG-motif and CGTCA-motif (30%) respectively. These results imply that different type of *cis-elements* in the same types of *PbbZIP* genes might have various functions (Table S7). Further, TCA-element involved in salicylic acid (SA) response, TGACG-motif and CGTCA had involve in as a methyl jasmonate (MeJA) responsive *cis-*elements and ABRE had abscisic acid (ABA) responsive *cis*-elements. These hormones (ABA, SA and MeJA) were involved in senescence and fruit ripening [56, 57]. These results suggesting that bZIP family may be associated with hormonal changes and involved in pear fruit senescence, ripening and maturation.

### Expression patterns of *PbZIP* genes in different fruit development stages

**Fig. 7:**
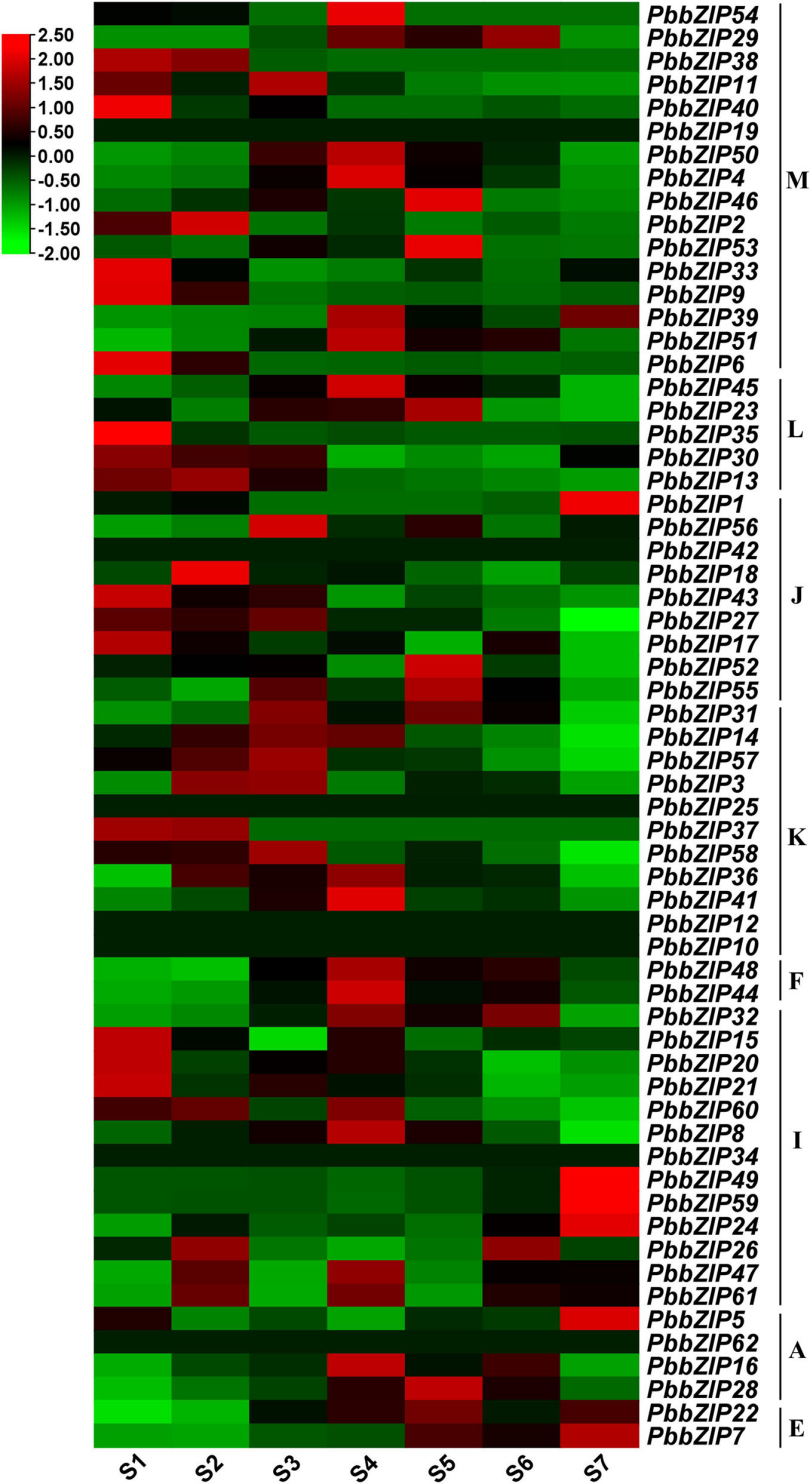
Heat map of *PbbZIP’s* gene family in fruit on different development stages 15DAB, 30DAB, 55DAB, 85DAB, 115DAB, mature stage, and fruit senescence stage) and Scale indicates the expression value

Based on RNA-seq data in pear fruit at different development stages, we investigate the expression profile of *PbZIP* genes (Table S8). The RNA-seq data carried out from previous studies conducted in 7 fruit development stages ((stage1-15DAB, stage2-30DAB, stage3-55DAB, stage4-85DAB, stage5-115DAB, stage6- mature stage, and stage7- fruit senescence stage). The microarray data was used to investigate the spatiotemporal expression profile of *PbZIP* members in different fruit stages. Fragments per kilobase million (FPKM) values were used to analyze the gene expression pattern. These results showed that *PbZIPs* expressed patterns increase and decreased in different development stages. *PbZIP49, PbZIP59* genes were abundantly expressed in stage-7 development stages, implying that maybe these genes play a critical role in fruit ripening and development. On the other hand, the expression profile of 2 genes (*PbZIP39, PbZIP51*) increased, and three genes (*PbZIP33, PbZIP6, and PbZIP9*) decrease during fruit development stages, suggesting that they may play an important role in early and later stages. The expression pattern in the development stage1 and stage2 is higher than that of other developmental stages of pear fruits (Fig. 7).

### Expression analysis of *PbZIP* genes under hormonal treatment

**Fig. 8:**
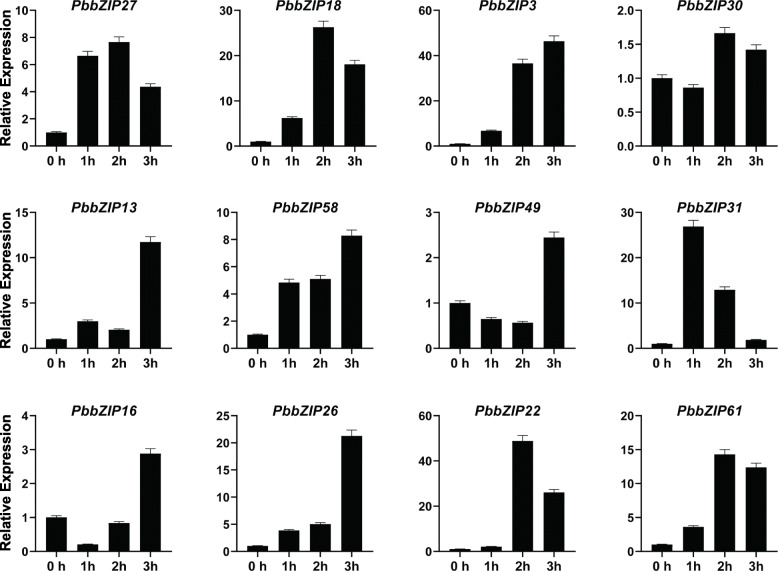
The relative expression patterns of *PbbZIP* genes in fruit under abscisic acid (ABA) hormonal treatments. Error bars show the standard error (SE) among three replicates

To further examine the expression pattern of *PbZIP* under hormonal treatment stresses (ABA, SA, and MeJA). Twelve genes were randomly selected after the spraying on pear fruit at different time duration (1 h, 2 h, 3 h). Finally, the expression patterns were analyzed using qRT-PCR. Application of exogenous hormonal spray two types of expression were observed inhibit and induced. Under the ABA (Abscisic acid) hormones treatment, most of the genes (*PbZIP3*, 13, 58, 49, 16, and 26) showed the highest expression pattern on 3 h, *PbZIP27*,18,30,22 and 61 genes peak expression on 2 h, and only one gene (*PbZIP31*) showed high expression as compared to control (CK) at 39 DAF. All genes showed significant expression under the ABA treatment (Fig. 8A). In SA (salicylic acid) treatment, we found that *PbZIP18*, 30, 13, 16, and 22 were achieved the highest expression pattern at 1 h. While *PbZIP27*, 3, 49, 26, and 61 strongly upregulated at 3 h. on the other hand, two genes (*PbZIP58* and 31) significantly expressed at 2 h (Fig. 8B). In case of MeJA (methyl jasmonate) treatment, genes have also seen the same trend as were in Abscisic acid and salicylic acid except for *PbZIP22*. Five genes, namely *PbZIP18*, 30, 49,16, and 61 upregulate expression at 3 h, while the *PbZIP22* gene inhibits expression at 1 h, 2 h and 3 h. The expression *PbZIP27*, 13, 31, and 26 significantly achieved expression at 1 h and 2 genes (*PbZIP58*, 3) at 2 h higher as compared to control. (Fig. 8C).

## Discussion

The basic leucine zipper (bZIP**)** transcription gene family has been identified and comprehensively studied in many plants such as *Arabidopsis*, *Oryza sativa*, *Zea mays*, *Glycine max, Coniothyrium minitans*, *Vitis vinifera*, *Ricinus communis L., Malus domestica*, *Fragaria vesca*, *Sorghum bicolor*, *B. napus*, *Solanum lycopersicum*, *B. distachyon*, *Prunus persica*, and evidence indicates that these genes participate in different physiological and developmental processes in plants. In this study, we identified and analyzed 62 genes in Chinese white pear and investigated their expression patterns on different fruit developmental stages under hormonal stress. Synteny analysis, evolutionary history, *cis*-elements analysis, chromosol location gene structure, and gene duplication were examined. Although the bZIP family has been identified and characterized in many crops, yet systematic and comparative study in pear remains unknown. However, bZIP genes were divided into 13 subfamilies (A, B(F/*Ara*), C(D/*Ara*), D, E (B/ *Ara*), F(H/*Ara*), G, H(E/*Ara*), I(I/Ara), J(G/*Ara*), K(A/*Ara*), L(C//*Ara*), M(S/*Ara*) based on the phylogenetic tree and Arabidopsis groups. A phylogenetic tree was constructed of bZIP genes by neighbor-joining methods (NJ-M). The evolutionary analysis of the tree showed that subfamily C, D, G, and H genes might be lost in the subfamily and expanded during the evolutionary mechanism in Chinese pear (Fig. 1). The number of bZIP genes in Arabidopsis, poplar, and *Prunus persica* was higher, while less than in gapes, apple, and strawberry as compared to a pear. These results indicate that the expansion of bZIP genes in pear. Different modes of gene duplication events (WGD-whole genome duplication, TD-tandem duplication, TRD-transposed duplication, and DSD- dispersed duplication) are the major source of driving force in the expansion and evolution process [58–60] for example, Hsf, and F-box gene family [61, 62]. Whole-genome duplication (WGD) can create a large number of duplicate genes in a short time [63]. In this present study, we identified four modes of duplication (DSDs, WGDs, TDs, and TRDs) in the PbbZIP gene family, 42 gene pairs in whole-genome duplication, 41 pairs in dispersed duplication, 2 tandem duplication, and 2 transposed duplications. In addition, WGD and DSD genes were involved in high proportion, indicating that these modes (WGD and DSD) may play a critical role in the evolution and expansion of *PbbZIPs*.

Furthermore, the pairwise synonymous (Ks) and non-synonymous (Ka) values were calculated in paralogous gene pairs, as shown in Fig. 4 and Table S5. The pairwise ka/ks value indicates that three types of selection (positive, purifying selection, and neutral selection). Ka/ks value of mostly paralogous genes indicates that purifying selection in *PbbZIPs* gene family. Tandem duplication events contained only two gene pairs, indicating that tandem duplication was not an important role in the expansion of *PbZIP* genes as compared to dispersed duplication (Table S5). Additionally, we analyzed the orthologous syntenic relationship between *Pyrus bretschneideri*, *Fragaria vesca, Prunus mume,* and *Prunus persica*. *Pyrus bretschneideri* and *Fragaria vesca* have 63 orthologous gene pairs, followed by *Pyrus bretschneideri* and *Prunus mume* have 61 orthologous gene pairs, while *Pyrus bretschneideri* and *Prunus persica* contained 72 orthologous gene pairs. These findings of collinearity analysis indicate that Rosaceae species (pear, peach, apple, and strawberry) strong similar syntenic orthologous and evolutionary relationship. Subsequently, the organization of gene structure (intron/exon) might imply that evolutionary trajectory in different bZIPs [64]. The amount of introns determines the plants potential to adapt and the development process [25].. Furthermore, the exon-intron compositions of bZIP individuals from the same group are frequently identified, as shown in Fig. 3. In certain gene families, this phenomenon is considered an evolutionary imprint, resulting in the development of functionally different paralogs [65, 66]. Those genes that have shorter introns or without introns created an activation role in evolutionary selection [67]. Some *PbbZIP* genes, especially *PbbZIP11*, 40, 19, 4, and 46, were prevalently lack of introns (Fig. 3), which could reduce the posttranscriptional processes for response to multiple abiotic stresses [68]. In this study, a maximal number of introns/exons (18/19) and minimal intron/exon (1/1) (Fig. 3). Moreover, 20 motifs were detected in *PbbZIPs* through the MEME webtool. The composition of motifs was shown in Table S4. These conserved motifs composition and different numbers indicate that the functional divergence between different subfamilies. Highly conserved motif 2 was distributed in most of the genes. These results suggest that conserved motifs may play various roles in determining specific functions of bZIP proteins [25].

**Fig. 9:**
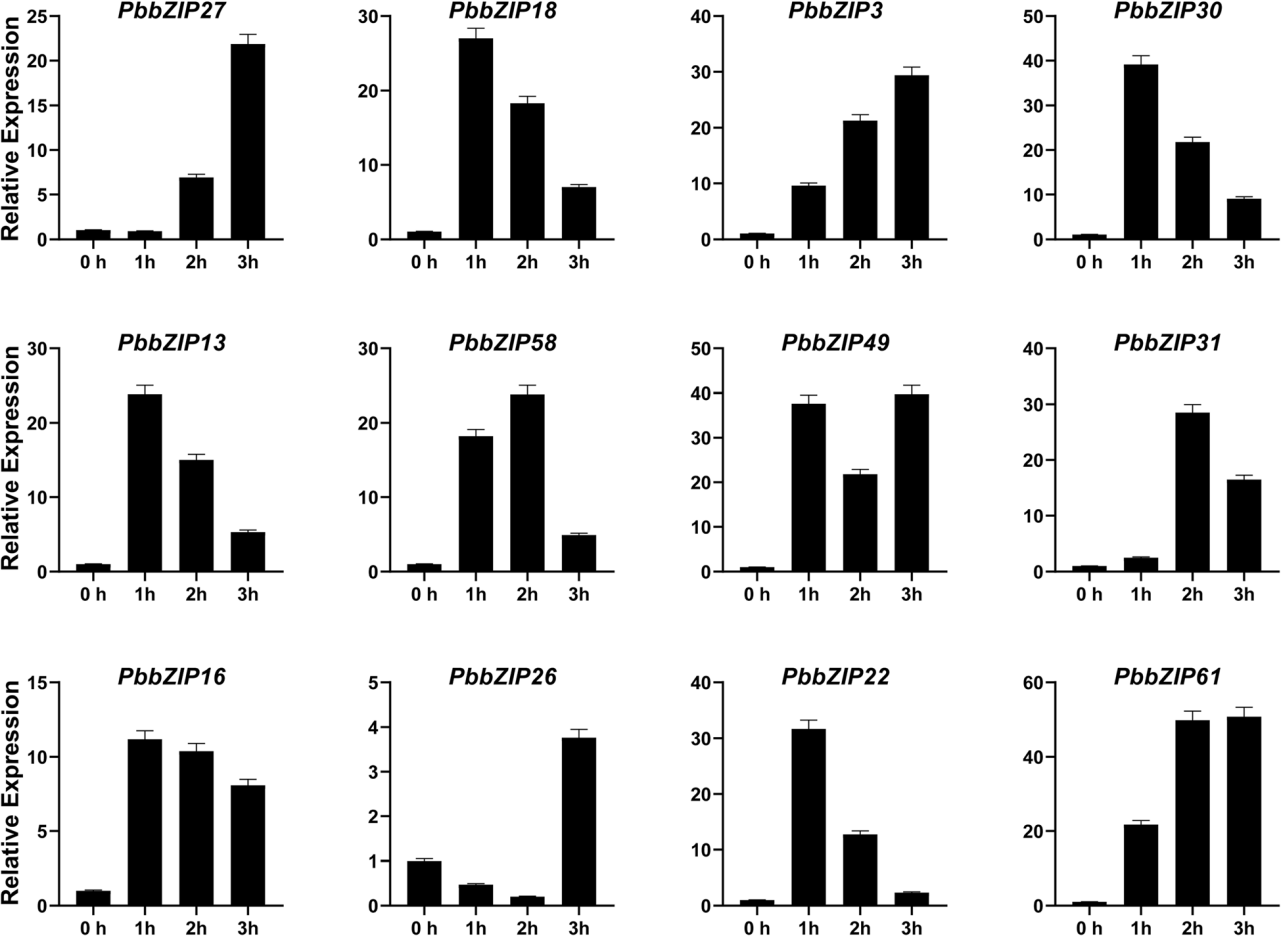
The relative expression patterns of *PbbZIP* genes in fruit under salicylic acid (SA) hormonal treatments. Error bars show the standard error (SE) among three replicates

**Fig. 10:**
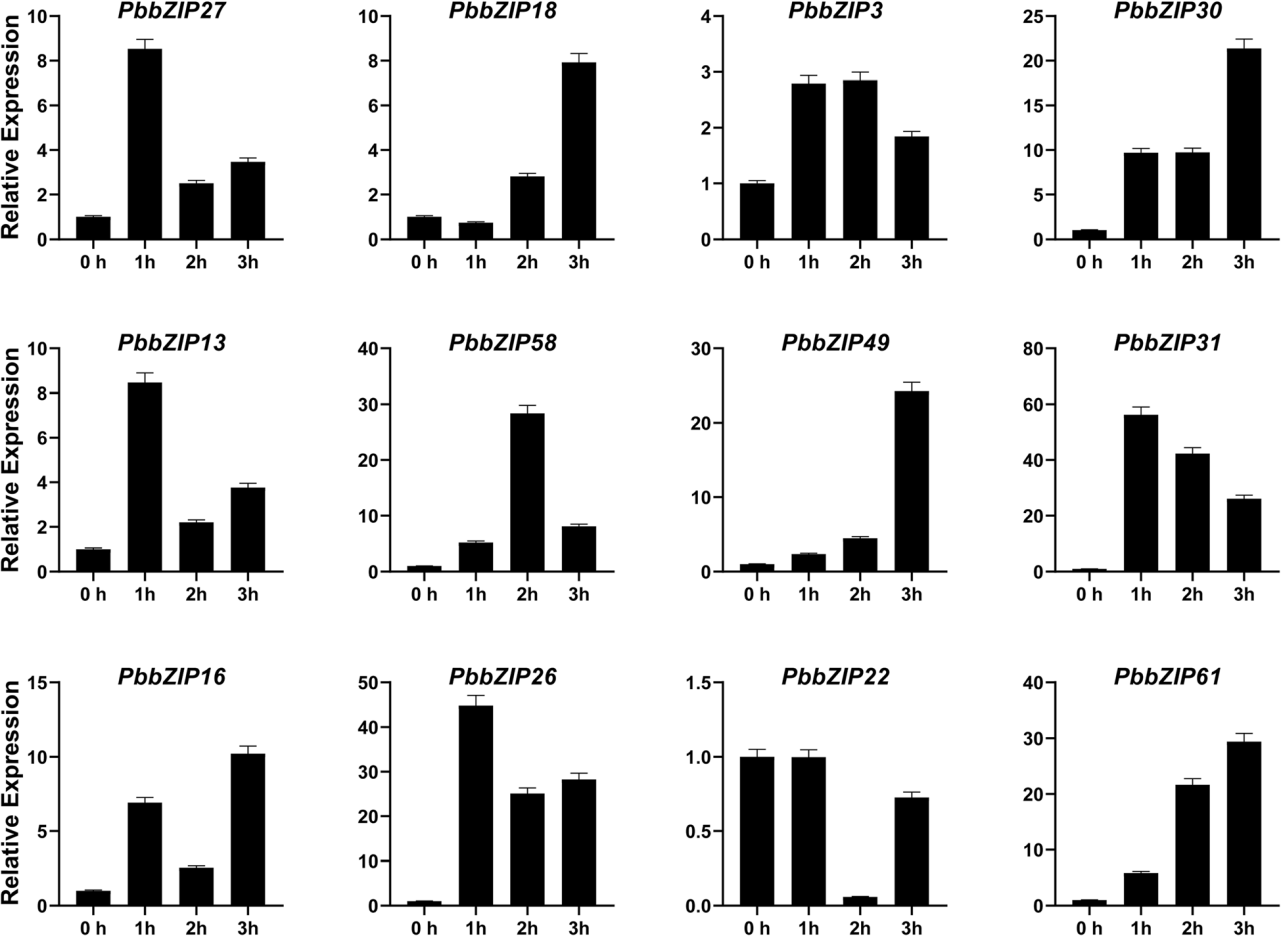
The relative expression patterns of *PbbZIP* genes in fruit under methyl jasmonate (MeJA) hormonal treatments. Error bars show the standard error (SE) among three replicates

Varieties of *cis-*elements were divided into three groups; 1) related to stress (LTR, G-box, and ARE), 2) related to phytohormones (GARE motif, TGACG motif, P-box, ABRE, and CGTCA-motif), and 3) related to plant growth/development (Box 4, CAT-box, 02-site) has been carried out on the promoter regions of *PbbZIPs*. Transcription factors are involved in stress response, for example, ABA [69], ethylene [35], light signaling [37, 38], hormones signaling [34, 35, 36], drought [46], cold stress [41, 42] and mechanical and osmotic stress [43]. OsABF2 is positively regulating Abiotic stress and ABA signaling in rice [70]. *SlbZIP38* gene identified in tomato is a response to salt stress [71]. *ZjbZIPs* play roles in development under abiotic and biotic stresses [5]. 10 TGA family genes in Arabidopsis play important roles in the SA defense signaling pathway and necrotizing pathogens [5, 72]. Plants improve their biotic and abiotic response after exogenous application of gibberellin, ethylene, SA, and ABA [73]. Proteins homologous to the FD (follower locus D; *ATbZIP14*) and FDP (FD paralog; *ATbZIP27*) transcription factors in *A. thaliana* were found in the FD-like clades, and they may play important roles in blooming. Finally, the qRT-PCR analysis was used to determine the expression profile of *PbbZIPs* in fruit under exogenous hormonal stress (ABA, SA, and MeJA). *PbbZIPs* genes significantly showed peaked expression, except one gene (*PbZIP22*) on different time duration in pear fruit (Fig. 8). Based on RNA- seq data, stage1 and stage4 genes are highly expressed. bZIP genes in many plant species, including tomato [74], apple [75], and watermelon [76], have been involved in fruit development and the ripening process. However, whether bZIPs are involved in Chinese pear fruit development and post-harvest ripening is unknown. In this present study, we found that *PbbZIP* genes were abundantly expressed on different development stages. These results imply that *PbbZIPs* genes are extensively involved in fruit development and ripening processes in Chinese white pear Figs. 9 and 10.

## Conclusion

In conclusion, A total of 62 *PbbZIPs* were identified in Chinese white pear, based on phylogenetic evolution, conserved motifs, and introns/exons analysis. *PbbZIPs* genes were categorized into 13 subfamilies. Whole-genome duplication (WGD) and dispersed duplication (DSD) might be highly contributed to the expansion of *PbbZIPs*. Moreover, chromosomal location, subcellular localization, synteny analysis, characteristics, calculate of ka/ks value, promoter regions, gene structure analysis. Finally, qRT-PCR results showed that *PbbZIPs* had a significant role in abiotic and biotic stress. Our results provide us a strong base for *PbbZIPs* roles in fruit development, ripening process, molecular mechanism, and evolutionary relationship, especially under multiple stress conditions.

## Methods

### Plant material and treatments

The fruit samples 39 days after flowering were collected from a 42-year-old pear plant, which was grown in a research horticulture orchard in Dangshan, Anhui, China. The exogenous hormonal sprayed on the whole surface of fruits with 200 μM salicylic acid (SA), 500 μM abscisic acid (ABA), and 500 μM Methyl jasmonate (MeJA) according to a previously described method [50, 77]. All fruit samples were harvested at 39 DAF and collected on 0 h, 1 h, 2 h, 3 h. Immediately all fruit samples were frozen in liquid nitrogen and transformed into − 80 °C for storage until further experiments.

### Identification, conserved domain, and sequence analysis

The whole-genome sequence of Chinese white pear along with GFF3 (general feature format file) and CDS (Coding sequences) was downloaded from the pear genome project online web server (http://peargenome.njau.edu.cn) [78]. Furthermore, the Arabidopsis bZIP protein sequence was downloaded from the TAIR web server (http://arabidopsis.org) and Blasted against the Chinese pear genome for carryout *PbbZIP* genes. Secondly, the Hidden Markov Model (HMM) of the bZIP (PF00170) domain was retrieved from the Pfam database (http://pfam.xfam.org) [79], and Blast was used for the extraction of bZIP transcription factors genes from the pear genome. HMMER v3.3.2 software package and NCBI web-based conserved domain (http://ncbi.nlm.nih.gov/structure/bwrpsb/pwrpssb.cgi) was performed with default parameters for the identification of integrity domain. Finally, all output candidate genes were verified using SMART (http://smart.embl-heidelberg.de) [80] and InterPro (htt://www.ebi.ac.uk/InterPro/search/sequence) [81].

### Gene structure and conserved motif

The MEME-suit (Multiple EM for Motif Elicitation) online tools (http://meme-suit.org) were visualized and identified the conserved motif with the following default parameters; the minimum width 6 residues and maximum width 50 residues and the maximum number of 20 motifs were identified [82]. Subsequently, the introns/exons analysis of *PbZIP* genes was found through general feature format (GFF3) files and visualized by using (GSDS v2.0) Gene Structure Display Server (http://gsds-gao-lab.org) [83].

### Chromosomal distribution and gene features

The information about starting and ending points of each bZIP protein was investigated from the pear annotation GFF3 files the position of genes displayed using MapChart v2.3 (http://www.wur.nl/en/shoe/Mapchart.htm) [84]. Afterward, the molecular weight (MW), Isoelectric point (pI), and length of the amino acid were computed using the online web tool ExPasy (http://www.expasy.org) [85].

### Gene duplications, synteny analysis, and calculation of ka/ks value

we used the Multiple collinearity Scan toolkit (MCScanx) [86] to examine the four modes of gene duplication (WGD, TD, TRD, and DSD) in *PbbZIP* genes and the synteny relationship between *Pyrus bretschneideri*, *Fragaria vesca, Prunus mume,* and *Prunus persica*. Finally, gene duplication and collinearity relationship displayed using circos software and Tbtool software [87] with default parameters. The ka/ks values were determined for the measurement of selection pressure during the expansion and evolution process of different gene pairs. The ka/ks values were obtained using ka/ks calculator (http://github.com/qiaoxin/scripts_for_Gb/tree/master /calculator_ka_ks_pipeline) [88].

### *Cis*-element analysis and gene ontology

The potential *cis*-regulatory elements on promoter regions of bZIP genes were analyzed. The promoter sequence of each bZIP was used, which is already downloaded from the pear genome project (http://peargenome.njau.edu.cn). Furthermore, the plantCare [89] web tool (http:///bioinformatics.psb.ugent.be/webtool/plantcare) to identify the putative *cis*-regulatory elements on promoter region. In the predicted genes, GO annotation and subcellular localization were determined by using the CELLOGO tool [90].

### Phylogenetic tree

A multiple protein sequence alignment (75 Arabidopsis, 86 poplar, 55 grapevine) was aligned with the full-length amino acid of bZIP members using MEGA-X software (http://www.megasoftware.net) [91]. A phylogenetic tree was constructed with neighbor-joining methods (NJ-M) with 1000 bootstrap replicates, the pairwise deletion and Poisson correction distance method were selected with others default parameters. On the other hand, alignments were used to construct a phylogenetic tree with a maximum likelihood method using the online IQ-TREE program. Finally, the phylogenetic tree was visualized using itol software (https://itol.embl.de/) [92].

### Insilico expression analysis

To investigate the expression patterns of *PbbZIP* genes in different fruit development stages (stage1-15DAB, stage2-30DAB, stage3-55DAB, stage4-85DAB, stage5-115DAB, stage6- mature stage, and stage7- fruit senescence stage) of *Pyrus bretschneideri* and FPKM value were carried from RNA-seq SRA (sequence read archive) data were downloaded from NCBI (http://www.ncbi.nlm.nih.gov) with following accession number, SRX1595645, SRX1595648, SRX1595646, SRX1595647, SRX1595651, SRX1595650, SRX1595652. Finally, the heat map of the *PbbZIP* family was visualized by using TBtool software [87].

### RNA isolation and quantitative real-time PCR analysis

Total RNA was isolated from pear fruits using RNA-prep plant pure kit (Tiangen - Beijing). The qRT-PCR specific primers were designed by using the Beacon Designer 7 software and a specific primer sequence as shown in Table S1. The total qRT-PCR mixture was 20 μl including 10 μl SYBER premix Ex Taq II, 1 μl forward, 1 μl reverse primer, 6 μl RNAase free water, and 2 μl cDNA. The pear tubulin gene was used as an internal control (Table S1) [78, 80]. The final expression patterns of *PbbZIP* genes were calculated using 2^−ΔΔCt^ methods [93].

## Supplementary Information

**Additional file 1: Table S1.** Primer of *PbbZIP’s* genes used for qRT-PCR.

**Additional file 2: Table S2.** Molecular characterization of *PbbZIP* genes Family in Chinese pear genome.

**Additional file 3: Table S3.** Predicted Molecular function, cellular components biological process, and subcellular localization of *PbbZIP* gene family in Chinese pear.

**Additional file 4: Table S4.** Motif sequence in bZIP genes.

**Additional file 5: Table S5.** Modes of gene duplication events, and non-Synonymous (ka) and synonymous (ks) values in *PbbZIP* genes Family.

**Additional file 6: Table S6.** List of bZIP orthologous gene pairs identified in *Fragaria vesca Prunus mume* and *Prunus persica.*

**Additional file 7: Table S7.** investigation of *cis*-acting elements in *PbbZIP’s* gene.

**Additional file 8: Table S8.** RNA-seq data of *PbbZIP’s* gene on different development stages of pear fruit.

**Additional file 9: Fig. S1.** Chromosomal locations of *PbZIP* genes in *P. bretschneideri*.

**Additional file 10: Fig. S2.** The phylogenetic tree of bZIP genes in *P. bretschneideri*, *F. vesca, P. persica,* and *M. domestica* with maximum likelihood method. Different color indicates different subfamilies (A-I).

## Data Availability

The Chinese white pear bZIP protein sequences were collected from the pear genome project (http://peargenome.njau.edu.cn). The Arabidopsis bZIP protein sequences were downloaded from the genome sequences of the Arabidopsis information source (TAIR) database (http://www.arabidopsis.org). All bZIP transcription factor genes in grapevine (*Vitis vinifera*) and poplar sequence were downloaded from the latest database version (V1) of the 12X assembly of grapevine genome (http://genomes.cribi.unipd.it/) and phytozome respectively. All RNA-Seq data were deposited in the NCBI SRA database under the following accession number, SRX1595645, SRX1595648, SRX1595646, SRX1595647, SRX1595651, SRX1595650, SRX1595652.

## Abbreviations

bZIP: basic leucine zipper
SA: salicylic acid
ABA: abscisic acid
MeJA: methyl jasmonate
HMM: Hidden Markov Model
DAF: Days after flowering
DAB: day after blooming
GO: gene ontology
NJ: neighbor-joining
GSDS: gene structure display server
TF: transcription factors
MEME: Multiple Em for Motif Elicitation
MW: molecular weight
pI: isoelectric point
MCSscanX: multiple collinearity scan toolkit
FPKM: Fragments per kilobase million
CDS: coding sequence
RNA: ribonucleic acid
qRT-PCR: real-time quantitative reverse transcription PCR
gDNA: genomic DNA
