# Supplementary figures and images for: Genome-wide identification and characterization of bZIP transcription factors and their expression profile under abiotic stresses in Chinese pear (Pyrus bretschneideri)
Source: BMC Plant Biol. 2021 Sep 9;21:413. doi: 10.1186/s12870-021-03191-3 (PMC8427902; doi:10.1186/s12870-021-03191-3)

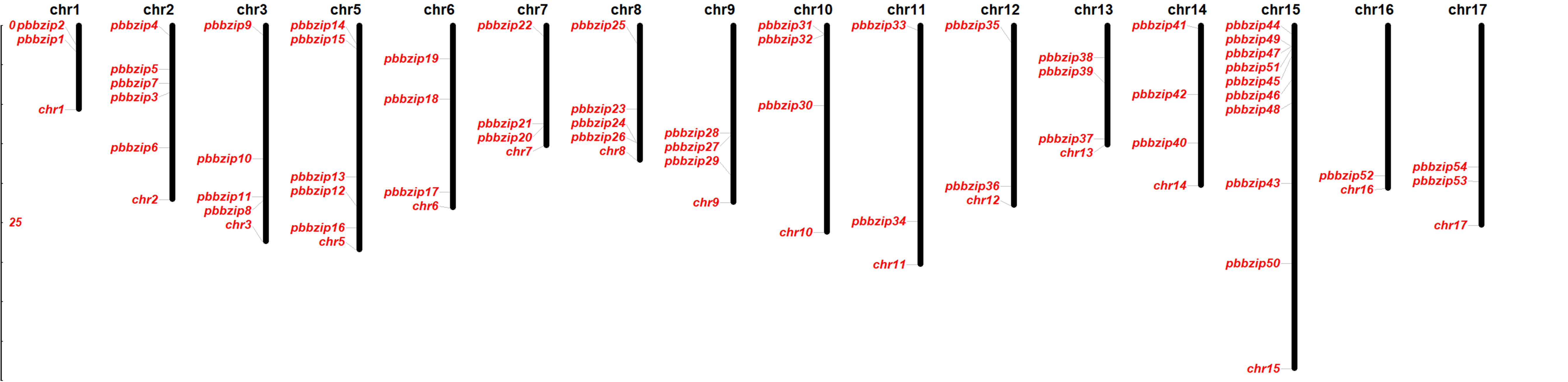

Supplement: Supplementary file 9 — Additional file 9: Fig. S1. Chromosomal locations of PbZIP genes in P. bretschneideri. [file 12870_2021_3191_MOESM9_ESM.pdf]

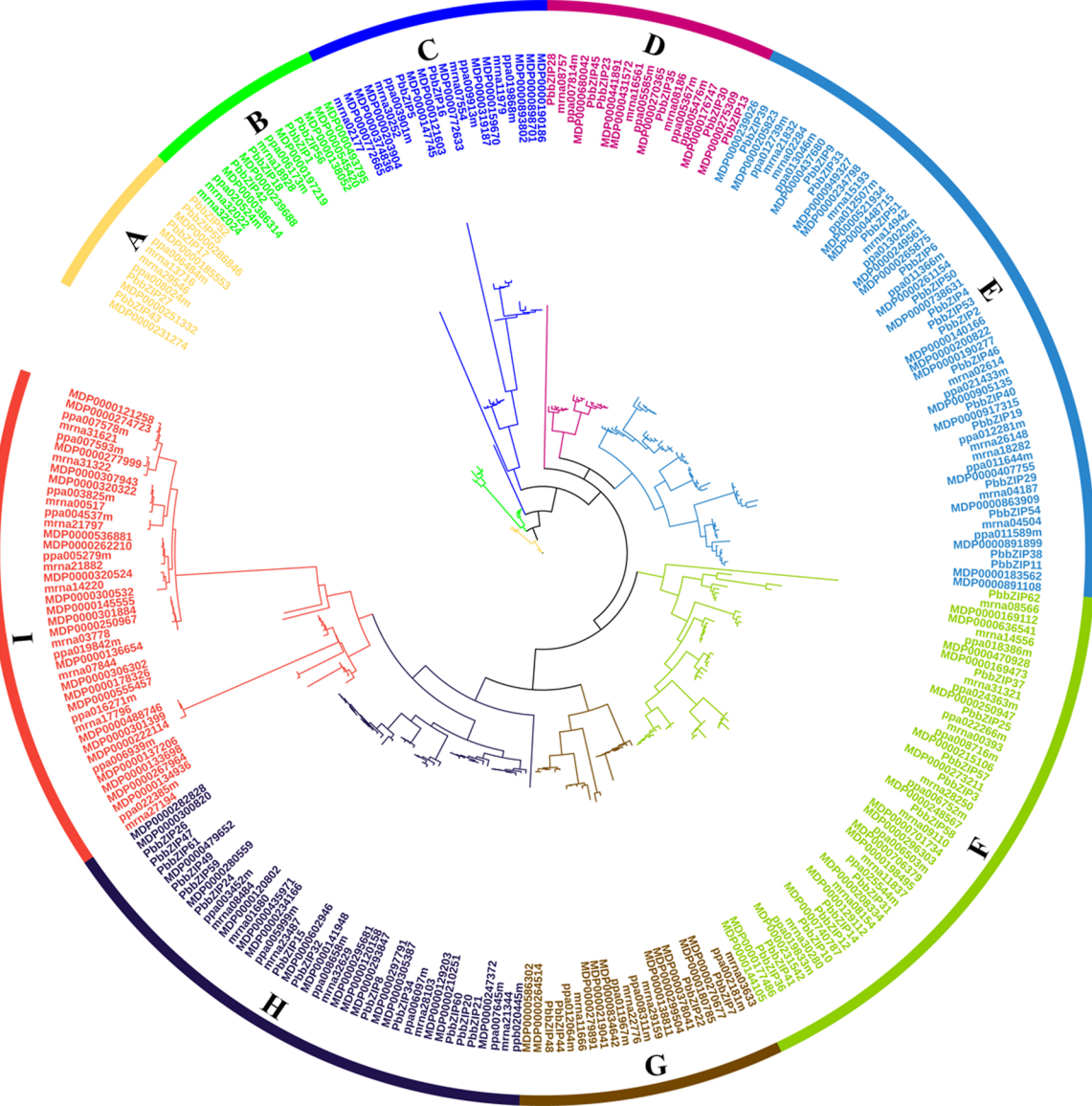

Supplement: Supplementary file 10 — Additional file 10: Fig. S2. The phylogenetic tree of bZIP genes in P. bretschneideri, F. vesca, P. persica, and M. domestica with maximum likelihood method. Different color indicates different subfamilies (A-I). [file 12870_2021_3191_MOESM10_ESM.pdf]
